# A High Plane of Nutrition Is Associated with a Lower Risk for Neonatal Calf Diarrhea on Bavarian Dairy Farms

**DOI:** 10.3390/ani11113251

**Published:** 2021-11-13

**Authors:** Ingrid Lorenz, Regina Huber, Florian M. Trefz

**Affiliations:** 1 Department Cattle Health Service, Bavarian Animal Health Service, 85586 Poing, Germany; 2 Veterinary Practice Mittermeier, 84559 Kraiburg, Germany; huber-regina@gmx.de; 3 Clinic for Ruminants with Ambulatory and Herd Health Services, Centre for Clinical Veterinary Medicine, LMU Munich, 85764 Oberschleißheim, Germany; f.trefz@med.vetmed.uni-muenchen.de

**Keywords:** calf diarrhea, risk factors, colostrum management, ad libitum feeding, iron supplementation

## Abstract

**Simple Summary:**

Calf mortality and morbidity are still unacceptably high on many dairy farms worldwide. Neonatal calf diarrhea is the most common cause of disease and death in young calves. This study attempted to identify risk factors that are associated with the outbreak of this multifactorial disease on Bavarian dairy farms. For this purpose, farms with calf diarrhea as a herd problem were compared to farms without veterinarian treatment for calf diarrhea for one year before the study visit. The main factor that was associated with a lower risk of neonatal diarrhea was the provision of adequate amounts of milk as compared with lower milk feeding levels. In contrast, supplementation with iron soon after birth was associated with a higher risk for calf diarrhea as a herd problem. It is well known that poor colostrum management and restricted milk feeding compromise calf development and weaken the immune system. Therefore, it is not surprising that calves receiving more colostrum and more milk have a higher chance of remaining healthy. Ad libitum feeding of calves in the first three weeks of life is recommended. The observed association between an increased calf diarrhea risk and supplementation with iron after birth requires further investigation.

**Abstract:**

In all bovine production systems, neonatal calf diarrhea remains worldwide an important issue of economic losses and animal welfare. The aim of the present study was to identify risk factors for neonatal calf diarrhea as a herd health problem on Bavarian dairy farms. For the purpose of this study, management factors related to calf health were retrospectively compared between 59 dairy farms with calf diarrhea as a herd problem with those of 18 control farms, where no veterinary treatment of calves for neonatal calf diarrhea took place for at least one year prior to the farm visit. A multivariable binary logistic regression analysis of management factors indicated that administration of 3 L or more of colostrum at the second feeding after birth (Odds ration [OR] = 0.21, 95% confidence interval [95% CI] = 0.05–0.89), ad libitum feeding of milk during the first week of life (OR = 0.06, 95% CI = 0.006–0.60), and administration of an iron containing preparation after birth (OR = 10.9, 95% CI = 1.25–95.6) were independently associated with the presence of a herd problem with neonatal diarrhea. Results of this study therefore suggest that a higher plane of nutrition is a protective factor with regard to the occurrence of neonatal diarrhea on Bavarian dairy farms. These findings support the establishment of ad libitum feeding programs in dairy calf rearing.

## 1. Introduction

Calf mortality and morbidity are still unacceptably high on many dairy farms worldwide. Even though heifer calf mortality decreased in the U.S. dairy industry throughout the past decades from 11 to 5% for calves born alive, still 33.8% of heifer calves experienced at least one disease event, with digestive disorders constituting the majority [1]. In a recent representative study of dairy farms in Germany, heifer calf mortality was found to be 3.7 to 7.4% depending on region, and treatment for calf diarrhea was performed in about 25% of calves [2].

Most commonly, infectious diarrhea in calves is caused by enterotoxigenic Escherichia coli, Cryptosporidium parvum, rotavirus, coronavirus, or some combination of these pathogens [3]. However, calf diarrhea is a multifactorial disease, which results from exposure to pathogens, on the one hand, and from deficiencies in management with impact on the calf’s immune status and/or the infectious pressure, on the other hand [4]. In a case-control study on Austrian dairy farms, variables significantly increasing the risk of diarrhea on farm were larger farm size, presence of other farm animals on the farm, placement of individual calf housing outdoors, and the presence of respiratory tract disease. Cleaning of the calving area after each calving decreased the risk for calf diarrhea [5]. In this study, no difference in colostrum and feeding management could be found between case and control farms. Even though it is widely recognized that colostrum management is the single most important management factor in determining calf health and survival [6], there are further studies that could either not find a correlation between failure of passive transfer and calf diarrhea [7,8] or that did not distinguish between causes of morbidity [1]. 

Another factor gaining increased recognition in recent years in the context of calf health and development is the plane of nutrition subsequent to the colostrum feeding. Meanwhile the beneficial effects of biologically normal milk feeding programs (also called intensified or accelerated feeding) were identified in numerous studies, which have been reviewed recently [9]. In addition to the obvious increased body weight and body growth, enhanced organ growth and development (e.g., rumen, small intestine, and mammary gland) as well as stimulation of the endocrine pancreas have been found. Metabolic changes include greater systemic metabolic activity and elevated metabolic activity in the ruminal epithelium and in the omental adipose tissue. Analogous to the effect of colostrum, a high plane of milk feeding is necessary to stimulate the somatotropic axis and to enhance maturation of the intestinal immune system [9]. Unsurprisingly, all these benefits also translate into lower mortality and prevalence of diseases in calves on a higher or biologically normal plane of nutrition in comparison to traditional restricted feeding [10,11,12,13,14].

The dairy industry in Bavaria is characterized by small structures with a mean number of 42 dairy cows per farm (November 2020) in mostly family run operations [15]. As a consequence, calf housing and practices of calf rearing are very diverse, leading to the assumption that it would be easier to identify risk factors for calf diarrhea, than it would be in larger operations with similar standard operating procedures. The veterinarians of the Bavarian Animal Health Service are regularly called by farm owners to assess calf management or investigate calf health problems. The objective of the present study was therefore to retrospectively analyze data retrieved from those farm visits to define risk factors for calf diarrhea as a herd problem in a case-control study.

## 2. Materials and Methods

Between January 2017 and April 2017, 14 veterinarians of the Bavarian Animal Health Services conducted 77 farm visits on dairy farms focusing on calf health and management as requested by the farm owners. During this period of time, the Bavarian Animal Health Service offered “calf health monitoring” visits in the framework of projects with the aim of improving farm animal health, partially funded according to the funding information provided below. All Bavarian farmers could take advantage of this opportunity. For this study, data derived from those visits were evaluated retrospectively (convenience sample).

According to farm owners, neonatal calf diarrhea as a herd health problem (including need for veterinary treatment) was present on 59 farms (Group P). On 18 farms no veterinary treatment of calves for neonatal calf diarrhea took place for at least one year prior to the farm visit. This does not mean that calf diarrhea did not occur, however, no treatment other than oral rehydration was performed. These farms served as a control group (Group C). To achieve 80% power at the 5% level of significance by assuming a 20% probability of exposure for a risk factor in the control group, this sample size was sufficient to detect an odds ratio of 5.3 (www.https://riskcalc.org/samplesize/, accessed on 18 October 2021). A standardized questionnaire was used to collect data during a face-to-face interview with the farm owner or manager. Areas of interest were farm characteristics, calving management, calf housing, and feeding, as well as hygienic measures. 

During the farm visit a 10 mL sample of blood was drawn from up to ten calves from 2 to 10 days old by venipuncture into a monovette without anticoagulant (Sarstedt AG & Co. KG, Nümbrecht, Germany). Samples were cooled and shipped by overnight courier to the laboratories of the Bavarian animal health services. Moreover, herd owners were asked to collect up to ten colostrum samples at the point of calf feeding, which is from the nipple of the feeding bucket or the tip of the esophageal feeder. Samples were immediately frozen on farm. Samples were collected on farm and transported frozen to the laboratories of the Bavarian animal health services. On arrival colostrum samples were stored at −24 °C until testing. 

Serum was separated from blood samples by centrifugation. Total protein (TP) was measured using an automatic analyzer (Konelab 30i, ThermoFisher Scientific, Waltham, MA, USA) by the biuret method. A threshold value of 58 g/L serum total protein was used to define good to excellent passive transfer [16]. 

Colostrum samples were thawed overnight at 4–8 °C, vortexed, and serially diluted 1:10 for 5 dilutions. Each dilution was plated on plate count agar for total plate count (TPC) and Water-blue Metachrome-yellow Lactose Agar acc. to GASSNER for total coliform count (TCC) in duplicate. Plates were incubated for 72 h at 30 °C, and 48 h at 37 °C, respectively. The number of colonies (cfu/mL) was recorded as the mean of the duplicate plates. According to McGuirk and Collins [17], total bacterial count should not exceed 100,000 cfu/mL, and fecal coliforms should be below 10,000 cfu/mL. Immunological quality of colostrum was estimated using Brix refractometry (PCE-032, PCE Instruments, Meschede, Germany). A threshold value of 22% Brix was used to define good quality colostrum [18].

Data were analyzed using IBM SPSS Statistics 24.0.0.1 and *p*-values < 0.05 were declared statistically significant. The association between the appearance of diarrhea on farm and binary or categorical variables was evaluated using a chi-square test or a Fisher’s exact test, if the expected frequency in one of the cells of the contingency table was less than five. Comparisons of continuous variables between case and control farms were made using a non-parametric Mann-Whitney U test, because most of those variables were not normally distributed as indicated by the results of a Shapiro-Wilk test. Variables with a *p*-value ≤ 0.2 that were considered relevant from a medical point of view were subsequently entered into a multivariable regression model with calculation of odds ratios (OR) and associated 95% confidence intervals (95% CI) using a stepwise backward procedure with a Wald *p* < 0.05 as selection criterion and using presence of diarrhea problems on farm as a binary outcome variable. If two variables were closely correlated to each other (*r_s_* > 0.6 or <−0.6), only that variable was entered into the model that had the lowest *p*-value in the preliminary univariable analysis. The fit of the final logistic regression model was evaluated by means of the Hosmer-Lemeshow Goodness-of-Fit test.

## 3. Results

The distribution of general farm characteristics was mostly similar between groups as can be seen in Table 1. Herd size was the same (Median = 75 dairy cows), however, German Fleckvieh was significantly more often kept by control farms. Furthermore, the milk yield was lower, and the calving interval was longer on problem farms. 

Calving pens were used on 78% of Group P farms and 61% of Group C farms. In three Group C farms and four Group P farms calvings took place in separate tie stalls, even though the cows were kept in cubicle houses. On 65.2% of problem farms with calving pen each calving took place in the calving pen as opposed to 100% on control farms. Further data on calving management are summarized in Table S1 of the Supplementary material.

Details on the colostrum management are presented in Table 2. There was no difference regarding immunological and hygienic quality of colostrum between groups. Vaccination of the dams against common pathogens for calf diarrhea was performed on half of the farms in both groups. Timing and volume of first colostrum feeding did not differ, however, calves on problem farms did significantly more often receive less than two liters of colostrum at the second feeding as opposed to three liters or more. The percentage of calves with total protein concentrations below 58 g/L was only numerically higher on control farms and the median serum total protein concentration was identical between groups.

Table 3 lists details of the feeding management subsequent to colostrum feeding. Whole milk was the preferred feeding source on all farms, milk replacer was only fed on 32.2 and 22.2% of Group P and Group C farms, respectively. Feeding of acidified milk and feeding of waste milk did not differ between groups. Calves on Group C farms did significantly more often receive their milk feed from their individually assigned feeding bucket than Group P calves. Control farms used significantly more often ad libitum feeding, and the calves on control farms did more often ingest more than 3 L of milk per meal than calves on problem farms. Calves on problem farms did more often only ingest roughage as additional feed from the second week, whereas calves on control herds received more often calf total mixed ration (TMR).

Information on the housing of calves can be found in Table S2 of the Supplementary material. The only difference that could be found on housing management between groups was that calves on Group P farms were more often housed close to adult cattle (*p* = 0.042). Calves on Group P farms were also prone to further childhood diseases, however, a statistical difference could only be found with regards to diarrhea in older calves (Table S3 in Supplementary material). The use of preventive measures (Table S4 of supplementary material) was very equally distributed between farms with the exception of the supplementation of iron after birth, which was numerically more often given on Group P farms.

Table 4 lists the variables that were finally entered into the multivariate regression model. Calves allowed to suckle the dam, ad libitum feeding after the first week, and separate feeding bucket for each calf after the first week were excluded from entering the model due to close correlation with the variables of newborn calves with dams longer than 3 h, ad libitum feeding in first week of life, and separate feeding bucket for each calf in first week of life.

After stepwise backward elimination three variables remained in the final model, as can be seen in Table 5. Variables remaining in the final model were 3 L or more at second feeding and ad libitum feeding in first week of life, which was associated with a lower risk for calf diarrhea as a herd problem. Furthermore, administration of an iron containing preparation after birth was associated with a higher risk of neonatal diarrhea as a herd problem.

## 4. Discussion

This study used a convenience sample of dairy farms that were visited by veterinarians of the Bavarian Animal Health Service to perform a calf management and health investigation. This was the major limitation of the study, because the visited farms were neither representative for dairy farms in the area, nor were the control farms randomly chosen and matched to the problem farms. Cow number was still the same between groups but considerably higher than the average cow number on Bavarian dairy farms of 39 in 2017 [15]. In a recent case-control study on Austrian dairy farms, larger farms had a higher risk of calf diarrhea problems, even though it was attempted to match case and control farms by herd size according to the study protocol [5]. The structure of the dairy industry is similar between Austria and Bavaria with small family-run businesses but with a tendency of farms to increase cow numbers. It is feasible that larger farms have more problems with calf diarrhea either due to an increase in workload but not in manpower or because calf facilities were not adapted to growing cow numbers. The reason that in this study no difference in cow numbers between groups was found lies probably in the fact, that more progressive well-managed farms would be more interested in the offer of having their calf management checked even without having obvious problems. This assumption is also supported by a higher milk yield and a shorter calving interval on control farms. 

A further limitation was the small number of farms, especially in the control group. Since this study relied on a convenience sample, it was unfortunately not possible to match farms. However, the only difference in farm structure was the breed distribution. It can be discussed whether this is due to selection bias, or if farms with breeds other than German Fleckvieh have indeed a greater risk for calf diarrhea problems. Svensson et al. [19] found a difference in calf diarrhea risk between breeds of calves; however, they did not discuss possible causes for this observation. Calf mortality was higher in German regions where mainly German Holstein or Red Holstein were kept in comparison to regions were the dual-purpose breed German Fleckvieh was the primary dairy breed [2]. Since calf diarrhea is a major cause of death in young calves, it can be assumed that there is also a difference between the main breed on farm and the incidence of diarrhea. One reason that is discussed is the difference in the economic value of the calves, which could lead to farmers neglecting calf management [2]. However, this is unlikely to explain the difference between breeds in the current study, since the farmers were obviously unhappy with the situation and seeking help, which makes it unlikely that they did not care for their calves for economic reasons. Another explanation could be that farms with Holsteins or mixed breeds may have recently increased cow numbers, which could explain a higher infectious pressure on the calves.

The availability of a calving pen did not differ between groups; however, there was a difference in the usage of the calving pen, if present. While all of group C farms indicated that they used the pen for each calving, the same was only the case in two thirds of problem farms. In the literature, contradicting evidence can be found concerning the role of calving pens for the risk of calf diarrhea. Frank and Kaneene [4] found that the use of individual calving areas and the removal of the bedding between calvings reduced the risk for calf diarrhea on farms with 50 to 99 cows, whereas Pithua et al. [20] could not find a difference between the use of single or multiple cow calving pens. Klein-Jöbstl et al. [5] found calving pens more often on problem farms than on control farms; however, the cleaning of the calving pen after each calving was identified as a preventive factor with regards to calf diarrhea as a herd problem. Taken together these findings indicate that neither the design nor the presence of a calving pen but the management thereof is an important factor for the prevention of calf diarrhea.

Serum IgG concentration as an indicator of the quality of the passive transfer of immunity is a clear predictor of calf morbidity and mortality [1]; however, several studies failed to make a clear connection between measures of colostrum management and risk of calf diarrhea [5,7,8]. In the current study, there was no difference between groups concerning the hygienic and immunological quality of colostrum or the serum total protein levels of the examined calves. In fact, in both groups there was a high percentage of calves with poor or fair passive transfer of immunity according to the definition of Lombard et al. [16]. This underlines the multifactorial nature of the disease and shows that optimal colostrum intake could differ between farms due to variations in husbandry conditions [21]. However, as the only definition for control farms was “no veterinarian treatment for calf diarrhea one year before the study visit” it cannot be ruled out that calf performance on control farms could be still improved by higher colostrum intake. The only difference found between groups was a higher volume of colostrum at the second feeding, given to calves on control farms. The latest research has shown that immunoglobulins are only one of many components that are of major importance for the calf, including the development of the intestinal mucosa and the maturation of the local immune system in the gut [22]. This is also promoted by extended feeding of colostrum or transition milk [23]. It is therefore possible that extended feeding of higher amounts of colostrum improved the calves’ resistance to calf diarrhea but did not have an impact on the serum total protein values due to gut closure. Another possible explanation could be that even though there is no close correlation (r_s_ > 0.6) between the volume at second feeding and other significant variables related to the further feeding of the calf, there is a likelihood that calves that receive a higher volume of colostrum at second feeding also will receive more milk further on. 

Calves on control farms were significantly more often fed ad libitum and more than 3 L per meal in the first week than calves on problem farms. Klein-Jöbstl et al. [5] could not find a difference between milk feeding volumes on farms with and without calf diarrhea problems; however, they did only distinguish between restricted and ad libitum feeding, and only 2 farms in each study group used ad libitum feeding. However, there is evidence that a higher plane of nutrition improves immune function [24] and also lowers mortality and the incidence of diarrhea and pneumonia [10,11,12] in calves. It is now recognized that biologically normal milk-feeding programs (ad libitum or close to ad libitum feeding in the first three weeks of life) do not only leave calves less hungry and thus improve calf welfare but also improve the systemic and gastrointestinal development of the calves [22]. The finding that two variables indicating a higher plane of nutrition reached statistical significance in the final multivariable regression model is therefore not surprising. A recent representative survey in the study area (Bavaria) has found that only on 10% of dairy farms is ad libitum feeding of calves practiced in the first weeks of life [2], while on U.S. dairy farms heifer calves were on average fed 5.6 L of milk or milk replacer per day [25], leaving a huge potential for improving calf health and performance in both dairy industry systems. 

An interesting finding was the observed association between an increased calf diarrhea risk and the supplementation of iron after birth. In contrast to findings of the present study, there are studies indicating a higher risk of calf diarrhea in calves with low iron levels [26] or an increased risk of iron deficiency anemia in calves with diarrhea [27]. It is therefore possible, that on farms with diarrhea problems farmers are more likely to supplement iron as a preventive measure, however, if that was the case it could also be expected that other preventive measures (e.g., supplementation of selenium and vaccination of the dam) would also be performed more often, which was not the case. On the other hand, it has been elucidated in the past 20 years that a concomitant decrease in iron in serum/plasma during acute inflammation is triggered by Hepcidin. This antimicrobial-like peptide hormone regulates the iron uptake from the intestines as well as the distribution of iron between cells and the extracellular space. Based on the fact that numerous pathogens require iron for their own metabolism, this mechanism is seen as being a part of the innate immunity [28]. Moreover, iron concentration in colostrum and milk is naturally low, as opposed to most other physiologically important elements [29]. It is conceivable that this adaptation was established during evolution to protect newborn calves from enteral overgrowth of iron dependent microorganisms. This effect would be overturned especially by oral iron supplementation. Certainly, this is an area where more research is needed to clarify possible beneficial or detrimental effects of iron supplementation in newborn farm animals.

In conclusion, the central findings of this retrospective analysis of calf management factors suggest that a high plane of nutrition plays a key role in the prevention of neonatal calf diarrhea. These findings support the establishment of ad libitum feeding programs in dairy calf rearing.

## Figures and Tables

**Table 1:** Farm data from 59 dairy farms with (Group P) and 18 dairy farms without (Group C) neonatal calf diarrhea as a herd health problem.

Variable	Group P Median (Q_1_/Q_3_) * or n (%)	Group C Median (Q_1_/Q_3_) * or n (%)	*p*-Value
Number of cows	75 (62–108)	75 (70–100)	0.86
Number of heifers	40 (30–68)	55 (42–69)	0.08
Number of calves	30 (20–40)	25 (20–37)	0.81
Breed			
German Fleckvieh	33 (55.9%)	17 (94.4%)	0.00
German Holstein	3 (5.1%)	0 (0.0%)	
German Braunvieh	7 (11.9%)	1 (5.6%)	
Multiple breeds	16 (27.1%)	0 (0.0%)	
Milk yield (kg)	8300 (7663–8950)	9000 (8175–9500)	0.03
Milk fat (%)	4.2 (4.1–4.3)	4.2 (4.1–4.2)	0.64
Milk protein (%)	3.5 (3.4–3.6)	3.6 (3.5–3.6)	0.08
Somatic cell count (mL)	150,000 (120,000–176,500)	150,000 (132,500–180,000)	0.53
Calving interval (days)	382 (375–400)	372.5 (368–379)	0.01
Replacement rate (%)	25 (20–30)	28 (24–30)	0.26
Housing system			
Cubicle house	56 (94.9%)	17 (94.4%)	1.00
Other	3 (5.1%)	1 (5.6%)	
Ventilation			
Outdoor climate	33 (55.9%)	10 (55.6%)	0.98
Other	26 (44.1%)	8 (44.4%)	
Youngstock housed separately			
Yes	20 (33.9%)	4 (22.2%)	0.35
No	39 (66.1%)	14 (77.8%)	

* Q_1_/Q_3_ = Interquartile range.

**Table 2:** Colostrum management on 59 dairy farms with (Group P) and 18 dairy farms without (Group C) neonatal calf diarrhea as a herd health problem.

Variable	Group P Median (Q_1_/Q_3_) * or n (%)	Group C Median (Q_1_/Q_3_) * or n (%)	*p*-Value
Percentage of colostrum samples with Brix values below 22% Brix	66.7 (50.0–77.8)	60.0 (40.0–66.7)	0.24
Percentage of colostrum samples with Total bacteria count < 100.000 cfu/mL.	20.0 (9.3–50.0)	11.1 (0–22)	0.23
Percentage of colostrum samples with Total coliform count < 10.000 cfu/mL.	94.5 (67.5–100.0)	85.0 (60.0–100.0)	0.39
Dam vaccination prepartum (against rotavirus, coronavirus, *E. coli*)			
Yes	29 (49.2%)	9 (50.0%)	0.95
No	30 (50.8%)	9 (50.0%)	
Source of colostrum			
Calf’s dam only	57 (96.6%)	18 (100.0%)	1.00
Pool	2 (3.4%)	0 (0.0%)	
Esophageal feeder is used sometimes			
Yes	33 (55.9%)	12 (66.7%)	0.42
No	26 (44.1%)	6 (33.3%)	
Warming of colostrum (multiple selections possible)			
Not performed	20 (33.9%)	8 (44.4%)	
Water bath	26 (44.1%)	5 (27.8%)	
Immersion header	20 (33.9%)	7 (38.9%)	
Microwave	0 (0.0%)	1 (5.6%)	
Pasteurization	1 (1.7%)	0 (0.0%)	
First colostrum meal within 2 h of life			
Yes	43 (72.9%)	15 (83.3%)	0.54
No	16 (27.1%)	3 (16.7%)	
Volume of colostrum at first meal			
3 liters	19 (32.2%)	10 (55.6%)	0.08
2 liters or as much as calf drinks	40 (67.8%)	8 (44.4%)	
Volume of colostrum at second feeding			
3 liters or more	28 (47.5%)	15 (83.3%)	0.01
Less than 3 liters	31 (52.5%)	3 (16.7%)	
Percentage of calves with serum total protein concentration <58 g/L.	69 (50–100)	80 (50–100)	0.68
Serum total protein concentration (g/L)	5.3 (4.8–5.9)	5.4 (4.9–5.7)	1.00
Storage of fresh colostrum at 4 °C			
Yes	8 (13.6%)	6 (33.3%)	0.07
No	51 (86.4%)	12 (66.7%)	
Freezing of surplus colostrum			
Yes	48 (81.4%)	12 (66.7%)	0.20
No	11 (18.6%)	6 (33.3%)	

* Q_1_/Q_3_ = Interquartile range.

**Table 3:** Feeding management on 59 dairy farms with (Group P) and 18 dairy farms without (Group C) neonatal calf diarrhea as a herd health problem.

Variable	Group P Median (Q_1_/Q_3_) * or n (%)	Group C Median (Q_1_/Q_3_) * or n (%)	*p*-Value
Main feed source			
Milk replacer	19 (32.2%)	4 (22.2%)	0.42
Whole milk	40 (67.9%)	14 (77.8%)	
Feeding of acidified milk			
Yes	24 (40.7%)	7 (38.9%)	0.89
No	35 (59.3%)	11 (61.1%)	
Feeding of waste milk			
Yes	17 (28.8%)	6 (33.3%)	0.71
No	42 (71.2%)	12 (66.7%)	
Separate feeding bucket for each calf (first week)			
Yes	35 (59.3%)	16 (88.9%)	0.02
No	24 (40.7%)	2 (11.1%)	
Separate feeding bucket for each calf (later)			
Yes	33 (55.9%)	15 (83.3%)	0.05
No	26 (44.1%)	3 (16.7%)	
Automatic feeder			
Yes	2 (3.4%)	0 (0.0%)	1.00
No	57 (96.6%)	18 (100.0%)	
Number of feedings (first week)			
Ad libitum	3 (5.1%)	6 (33.3%)	0.00
2 or 3 meals per day	56 (94.9%)	12 (66.7%)	
Number of feedings (later)			
Ad libitum	4 (6.8%)	6 (33.3%)	0.01
2 or 3 meals per day	55 (93.2%)	12 (66.7%)	
Volume per feeding (first week)			
More than 3 L	10 (16.9%)	8 (44.4%)	0.02
3 L or less	49 (83.1%)	10 (55.6%)	
Volume per feeding (later)			
More than 3 L	27 (45.8%)	10 (55.6%)	0.47
3 L or less	31 (52.5%)	8 (44.4%)	
Additional feeding from second week on			
Roughage	45 (76.3%)	9 (50.0%)	0.04
Calf starter	22 (37.3%)	6 (33.3%)	0.78
Roughage and calf starter	20 (33.9%)	4 (22.2%)	0.35
Calf TMR	13 (22.0%)	10 (55.6%)	0.01
Water	47 (79.7%)	17 (94.4%)	0.17
Cleaning of nipple after each feeding			
Yes	29 (49.2%)	9 (50.0%)	0.95
No	30 (50.8%)	9 (50.0%)	

* Q_1_/Q_3_ = Interquartile range.

**Table 4:** Variables entered into a multivariate regression model with p-values from univariate regression.

Variable	*p*-Value
Calving pen cleaned after every calving	0.14
Newborn calves with dams longer than 3 h	0.08
3 L of colostrum at first feeding	0.08
3 or more liters of colostrum at second feeding	0.01
Ad libitum feeding during first week of life	0.00
Separate feeding bucket for each calf in first week of life	0.02
Housing of calves close to cows	0.04
Supplementation of iron after birth	0.16

**Table 5:** Final multivariate regression model with odds ratio (OR) and 95% confidence interval (CI) ^1^.

Variable	Regression Coefficient	Standard Error	Odds Ratio (OR)	95% Confidence Interval	*p*-Value
3 or more liters of colostrum at second feeding	−1.56	0.74	0.21	0.05–0.89	0.04
Ad libitum feeding during first week of life	−2.83	1.18	0.06	0.01–0.60	0.02
Supplementation of iron after birth	2.39	1.11	10.94	1.25–95.62	0.03

^1^ Hosmer-Lemeshow Goodness-of-Fit test: Chi-square: 0.72, df: 3. *p* = 0.87.

## Data Availability

The data presented in this study is available on reasonable request from the corresponding author.

## Supplementary Materials

The following are available online at https://www.mdpi.com/article/10.3390/ani11113251/s1, Table S1: Calving management on 59 dairy farms with (Group P) and 18 dairy farms without (Group C) neonatal calf diarrhea as a herd health problem. Table S2: Housing management on 59 dairy farms with (Group P) and 18 dairy farms without (Group C) neonatal calf diarrhea as a herd health problem. Table S3: Frequency of further calfhood diseases on 59 dairy farms with (Group P) and 18 dairy farms without (Group C) neonatal calf diarrhea as a herd health problem. Table S4: Preventive measures performed on 59 dairy farms with (Group P) and 18 dairy farms without (Group C) neonatal calf diarrhea as a herd health problem.
