# Supplementary material for: A High Plane of Nutrition Is Associated with a Lower Risk for Neonatal Calf Diarrhea on Bavarian Dairy Farms
Source: Animals (Basel). 2021 Nov 13;11(11):3251. doi: 10.3390/ani11113251 (PMC8614331; doi:10.3390/ani11113251)
Supplement: Supplementary file 1 [file animals-11-03251-s001.zip › animals-1419535-supplementary.pdf]

Supplementary material

Table S1: Calving management on 59 dairy farms with (Group P) and 18 dairy farms without (Group C) neonatal calf diarrhea as a herd health problem.

**Variable**	**Group P** Median (Q_1/Q_3)* or n (%)	**Group C** Median (Q_1/Q_3)* or n (%)	***p*-Value**
Calving pen			
Yes	46 (78.0%)	11 (61.1%)	0.16
No	13 (22.0%)	7 (38.9%)	
Calving pen used for every calving			
Yes	30 (65.2%)	11 (100%)	0.02
No	16 (34.8%)	0 (0.0%)	
Calving area cleaned after every calving			
Yes	10 (16.9%)	6 (33.3%)	0.14
No	49 (83.1%)	12 (66.7%)	
Calving area disinfected after every calving			
Yes	2 (3.4%)	1 (5.6%)	0.68
No	57 (96.6%)	17 (94.4%)	
Calving area never used for sick cows			
Yes	17 (28.8%)	5 (27.8%)	0.93
No	42 (71.2%)	13 (72.2%)	
Calving area always occupied			
Yes	20 (33.9%)	8 (44.4%)	0.09
No	39 (66.1%)	10 (55.5%)	
Newborn calves with dams longer than 3 hours			
Yes	17 (28.8%)	1 (5.5%)	0.07
No	42 (71.2%)	17 (94.4%)	
Calves allowed to suckle			

the dam			
Yes	16 (27.1%)	1 (5.5%)	0.09
No	43 (72.9%)	17 (94.4%)	

* Interquartile range

Table S2: Housing management on 59 dairy farms with (Group P) and 18 dairy farms without (Group C) neonatal calf diarrhea as a herd health problem.

**Variable**	**Group P** Median (Q_1/Q_3)* or n (%)	**Group C** Median (Q_1/Q_3)* or n (%)	*p*-**Value**
Individual housing of newborn calves			
Yes	49 (83.1%)	17 (94.4%)	0.44
No	10 (16.9%)	1 (5.6%)	
Pen (multiple selections possible)	37 (62.7%)	11 (61.1%)	
Hutch (multiple selections possible)	40 (67.8%)	13 (72.2%)	
Group housing			
Within first 2 weeks	18 (30.5%)	6 (33.3%)	0.82
With 3 weeks or more	41 (69.5%)	12 (66.7%)	
Management of group housing			
Continuous	51 (86.4%)	16 (88.9%)	1.00
All in – all out	8 (13.6%)	2 (11.1%)	
Calves housed close to adult cattle			
Yes	33 (55.9%)	5 (27.8%)	0.04
No	26 (44.1%)	13 (72.2%)	

* Interquartile range

Table S3: Frequency of further calfhood diseases on 59 dairy farms with (Group P) and 18 dairy farms without (Group C) neonatal calf diarrhea as a herd health problem.

**Variable**	**Group P** Median (Q_1/Q_3)* or n (%)	**Group C** Median (Q_1/Q_3)* or n (%)	***p*-Value**
Diarrhoea in calves older than 3 weeks			
<25% of calves	46 (78.0%)	18 (100.0%)	0.02
≥25% of calves	13 (22.0%)	0	
Respiratory disease			
<25% of calves	43 (72.9%)	17 (94.4%)	0.06
≥25% of calves	16 (27.1%)	1 (5.6%)	
Sucking weakness			
<25% of calves	47 (79.7%)	18 (100.0%)	0.06
≥25% of calves	12 (20.3%)	0	

* Interquartile range

Table S4: Preventive measures performed on 59 dairy farms with (group P) and 18 dairy farms without (group C) neonatal calf diarrhea as a herd health problem.

**Variable**	**Group P** Median (Q_1/Q_3)* or n (%)	**Group C** Median (Q_1/Q_3)* or n (%)	***p*-Value**
Oral vaccine against E. coli			
Yes	4 (6.8%)	0 (0.0%)	0.6
No	55 (93.2%)	18 (100.0%)	
Halofuginone			
Yes	18 (30.5%)	5 (27.8%)	0.8
No	41 (69.5%)	13 (72.2%)	
Preventive treatment			

against Eimeria spp.			
Yes	22 (37.3%)	7 (38.9%)	0.90
No	37 (62.7%)	11 (61.1%)	
Selenium and vitamine E supplementation			
Yes	24 (40.7%)	8 (44.4%)	0.78
No	35 (59.3%)	10 (55.6%)	
Supplementation of iron after birth			
Yes	20 (33.9%)	3 (16.7%)	0.16
No	39 (66.1%)	15 (83.3%)	

* Interquartile range
